# D-galactose causes sinoatrial node dysfunction: from phenotype to mechanism

**DOI:** 10.18632/aging.205196

**Published:** 2023-11-09

**Authors:** Heng Zhang, Chen Chen, Yue Liu, Wei Chen, Jing Qi, Yue Xu, Lu Ren, Guanlin Yang, Dongyu Min, Zhuang Liu, Xintong Cai, Miao Hao, Guanzhen Xu, Ping Hou

**Affiliations:** 1 Shandong Provincial Hospital Affiliated to Shandong First Medical University, Jinan 250021, China; 2 Shandong University of Traditional Chinese Medicine, Jinan 250355, China; 3 Shenyang Medical College, Shenyang 110034, China; 4 Affiliated Hospital of Liaoning University of Traditional Chinese Medicine, Shenyang 116600, China

**Keywords:** D-galactose, senescence, oxidative stress, sinoatrial node dysfunction, ion channels dysfunction

## Abstract

With the population aging, age-related sinoatrial node dysfunction (SND) has been on the rise. Sinoatrial node (SAN) degeneration is an important factor for the age-related SND development. However, there is no suitable animal modeling method in this field. Here, we investigated whether D-galactose could induce SAN degeneration and explored the associated mechanism. *In vivo*, twelve C57BL/6 mice were divided into Control and D-galactose group to receive corresponding treatments. Senescence was confirmed by analyzing the hair and weight; cardiac function was evaluated through echocardiography, cerebral blood flux and serum-BNP; the SAN function was evaluated by electrocardiogram; fibrotic change was evaluated by Masson's trichrome staining and oxidative stress was assessed through DHE staining and serum indicators. Mechanism was verified through immunofluorescence-staining and Western blotting. *In vitro*, mouse-atrial-myocytes were treated with D-galactose, and edaravone was utilized as the ROS scavenger. Senescence, oxidative stress, proliferation ability and mechanism were verified through various methods, and intuitive evidence was obtained through electrophysiological assay. Finally, we concluded that D-galactose can be used to induce age-related SND, in which oxidative stress plays a key role, causing PITX2 ectopic expression and downregulates SHOX2 expression, then through the downstream GATA4/NKX2-5 axis, results in pacing-related ion channels dysfunction, and hence SND development.

## INTRODUCTION

Sinoatrial node dysfunction (SND)/Sick sinus syndrome (SSS) is a disease characterized by sinoatrial node (SAN) pacing or conduction dysfunction caused by SAN or (and) its surrounding tissue dysfunction. It is often manifested by sinoatrial bradycardia, sinoatrial block, sinoatrial arrest and other arrhythmia, is one of the common causes of cardiogenic syncope and sudden death [1]. SND has been associated to the occurrence of senescence. The incidence rate of SND among people aged >65 years is estimated to be about 1/600, whereas the average occurrence age ranges between 73 and 76 years old [2]. Studies have shown that SND incidence gradually increases with age, and normal SAN function gradually declines during aging [3, 4]. Senescence-induced SAN degeneration is an important risk factor for age-related SND. Predictions from epidemiological studies in the United States indicate that senescence is expected to increase in the number of new SND cases from 78000 in 2012 to 172000 in 2060 [5, 6].

The reproduction of animal models poses a challenge to age-related SND research. Although some modeling methods have been efficiently applied in the field of SND studies, they are not completely suitable for age-related SND research. Studies have associated ischemia-reperfusion, chemical or physical methods with destruction of the SAN tissue and function [7, 8], despite their effectiveness, however, the associated injuries are not only irrelevant to senescence, but also cannot be used to simulate SAN degeneration and age-related SND. Angiotensin II injection through subcutaneous micro-osmotic pumps is a superior approach than direct SAN destruction [9]. Although this method can successfully induce SAN fibrosis, thereby allowing effective simulation of SAN degeneration, however, the approach has been found to cause obvious heart failure. Its application is also constrained by high cost of this disposable osmotic pumps, especially for some research groups with insufficient funds. Screening individuals with significantly lower heart rates from natural senescence animals has also been reported. However, the number of eligible animals obtained by this method remains low, thus this method cannot be applied in large-scale experiments. Other modeling methods, such as specific gene knockout, have also been employed, although this may only be applicable to specific studies and not for age-related SND.

Senescence is an inevitable life process regulated by various of complex mechanisms and factors. Among them, oxidative stress is not only a condition but also a result of senescence. According to the oxidative stress hypothesis, senescence results from a decline in the body’s antioxidant capacity, which leads to age-dependent impairment caused by reactive oxygen species (ROS) accumulation [10, 11]. The human body is an oxidation-antioxidant system, as the body ages, ROS gradually accumulates, thereby breaking the system’s balance and causing oxidation, a phenomenon that leads to senescence and development of age-related diseases. Senescence people are often characterized by higher oxidation levels, and are more likely to suffer from SND, while accumulation of ROS in the SAN is also an important mechanism of SSS.

**Figure 1:**
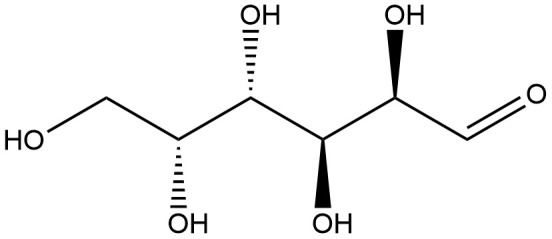
Structural formula of D-galactose.

D-galactose (Figure 1) is a monosaccharide that can be used to induce senescence in both animals and cells [12, 13]. Numerous studies have demonstrated that D-galactose can induce oxidative stress through various ways, thereby causing senescence. To date, however, whether D-galactose can induce senescence in the SAN remains unknown. In our previous study, we demonstrated that application of D-galactose not only successfully caused pulsatile dysfunction in human-induced pluripotent stem cell-derived cardiomyocytes (hiPSC-CMs), but also downregulated expression of *Shox2* and *Cav3.1* while inducing oxidative stress [14]. *Shox2* is a key gene controlling the development and differentiation of pacemaker (P) cells in the SAN, while *Cav3.1* encodes T-type calcium channels that play an important role in phase-4 automatic depolarization of P cells, which is of great significance for maintaining the autonomic rhythm of P cells. In the current study, we evaluated whether D-galactose could induce SAN degeneration via senescence in C57BL/6 mice, and explored the potential underlying mechanism *in vitro*. Our results are expected to provide novel insights for age-related SND.

## MATERIALS AND METHODS

### Animals

Twelve C57BL/6 mice (3-month-old, both genders) were randomly divided into 2 equal groups (n=6), namely Control and D-galactose. Mice in the D-galactose group were administered with D-galactose (200mg/kg/day) daily via subcutaneous injection on the back for 8 weeks to induce aging [15–17], whereas those in the Control group were given an equivalent volume of normal saline. The animals were weighed at week 1 and 8 after administration, and data on electrocardiogram, echocardiogram, and cerebral blood flux were obtained. Next, the animals were euthanized via intraperitoneal injection (i.p.) of sodium pentobarbital (150 mg/kg), and their hearts and blood were collected.

D-galactose (T0591, purity: 99.84%) was purchased from Topscience Medicine Technology (China), while C57BL/6 mice were purchased from Changsheng Biotechnology (China, Production License: SCXK (Liao) 2020-0001), and raised with no dietary restrictions.

### Electrocardiography and echocardiography measurement

Electrocardiography was used to record heart rate and rhythm, while echocardiography was employed to assess heart function. Briefly, mice were first anesthetized using sodium pentobarbital (30 mg/kg, i.p.), placed flat on the operating table, their limbs fixed, and electrodes subcutaneously inserted into the limbs to connect the electrocardiograph (MedLab-U/4C501H, Meiyi, China) to record heart rate and rhythm. Next, their chest hair was shaved, and a doppler ultrasound (Vinno 6 Vet, Vinno, China) was performed to assess cardiac function.

### Cerebral blood flux measurement

Cerebral blood flux measurement was performed to assess organ perfusion and heart function. In brief, mice were first anesthetized via sodium pentobarbital (30 mg/kg, i.p.), fixed on the operating table in a prone position, and their hair was shaved. The animals were then surface sterilized, the skin on the head incised to remove the periosteum and expose the skull. Next, a probe of laser speckle blood flowmeter (MoorFLPI-2, Moor Instruments, UK) was aligned at the center of the skull and the focus was adjusted to allow cerebral blood flux measurement.

### Brain natriuretic peptide and oxidative stress indicators assay

Brain natriuretic peptide (BNP) assay was used to evaluate heart function, whereas oxidative stress indicators superoxide dismutase (SOD) and malondialdehyde (MDA) assay were employed to quantify oxidative stress levels. Briefly, blood was collected from mice in each group into an EDTA-K2 anticoagulation tube, and serum was obtained through a 15-min centrifugation (4° C, 1000×*g*). Next, BNP, SOD and MDA assay kits were used to measure BNP, SOD and MDA concentrations, respectively, in serum, according to the manufacturer’s instructions.

BNP (SEKM-0151), SOD (BC0175) and MDA (BC0025) assay kits were purchased from Solarbio Biotechnology (China).

### Masson’s trichrome staining

Masson’s trichrome staining was used to label fibers, and collagen volume fraction (CVF) was calculated to analyze the degree of fibrosis. Heart tissues including around the SAN area were collected and paraffinized. They were subsequently cut into 4 μm thick serial sections (crosscutting). After deparaffinizing and washing, the sections were stained through Weigert’s iron hematoxylin for 10 minutes. This was followed by washing and staining with Masson’s composite staining for 10 minutes. Next, 2% glacial acetic acid and 1% dodeca molybdophosphoric acid solution were used to perform differentiation for 5 minutes. Next, they were stained with aniline blue for 5 minutes and then soaked in 0.2% glacial acetic acid without washing. This was followed by dehydration in 95% ethanol, and then by absolute ethanol. Finally, Xylene and a neutral glue were applied for clearing and sealing, respectively.

Masson’s trichrome staining kit (G1340) was purchased from Solarbio Biotechnology (China).

### Cell culture and si-RNA transfection

hiPSC-CMs and mouse atrial myocytes were used in *in vitro* studies. hiPSC-CMs were cultured in cardiomyocyte culture medium, while mouse atrial myocytes were cultured in Dulbecco’s modified Eagle’s medium (DMEM)/F12, supplemented with 10% fetal bovine serum and 1% penicillin/streptomycin. All cells were cultured in an incubator maintained at 37° C and 5% CO_2_ atmosphere (HeraCell 150i, Sanyo, Japan). The cells were divided into Control, D-galactose, Edaravone, si-NC and si-*Pitx2* groups. Cells in the D-galactose group were treated with D-galactose (10g/L) for 48 hours, while those in the Edaravone group were exposed to edaravone (40μM) for 1 hour, then co-incubated with D-galactose for 48 hours. Cells in the si-NC and si-*Pitx2* groups were transfected with si-NC and si-*Pitx2* gene interference sequence for 24 hours respectively, then co-incubated with D-galactose for 48 hours. For transfection, si-RNA was first diluted with riboFECT™ CP buffer, with riboFECT™ CP reagent, incubated for 15 mins at room temperature, then mixed with antibiotic-free medium to generate a transfection complex (working concentration of siRNA is 20nM). Next, 2 ml of the transfection complex was seeded in 6-well plates, and transfected for 24 hours.

hiPSC-CMs (HELP4111) and cardiomyocyte culture medium (HELP3001-2) were purchased from Help Stem Cell Innovations (China), mouse atrial myocytes (CL-0605) were purchased from Procell (China). DMEM/F12 medium (AG29749915) was bought from Hyclone (USA) whereas fetal bovine serum (11011-8611) was obtained from Sijiqing Biotechnology (China). Edaravone (T0407) was obtained from Topscience Medicine Technology (China), while si-RNA sequences (siG171225040450), riboFECT™ CP buffer (C10511-05) and riboFECT™ CP reagent (C10511) were acquired from Ribo Biotechnology (China).

### Immunofluorescence staining

For *in vivo* experiments, heart tissues including the SAN area were collected and paraffinized, and cut into 4 μm thick serial sections (crosscutting). The sections were dewaxed and dehydrated for antigen retrieval, blocked with 5% BSA for 1 hour, then incubated overnight with primary antibody against HCN4 (diluted 1:200) in a humid box at 4° C. Next, the sections were washed, then incubated for 1 hour with FITC-conjugated goat anti-rat IgG (diluted 1:500) at 37° C. The sections were washed again, stained with an anti-fluorescence quenching solution containing DAPI, then visualized under a fluorescence microscope (BX51TF, Olympus, Japan).

For *in vitro* experiments, mouse atrial myocytes were first seeded in a glass bottom cell culture dish. After culturing and treatment, the cells were fixed with 4% paraformaldehyde for 10 minutes, no permeabilization for HCN4/CAV3.1 assay to avoid damage to membrane proteins, in contrast, permeabilization with triton X-100 for 10 minutes for P16 assay to expose nucleoproteins. The specimens were washed with PBS, blocked with 1% BSA for 1 hour, then incubated overnight with primary antibodies against HCN4 (diluted 1:200) and CAV3.1 (diluted 1:200) or P16 (diluted 1:200) in a humid box at 4° C. The cells were washed, then incubated for 1 hour with TRITC-conjugated goat anti-rat IgG (diluted 1:50) and CoraLite488-conjugated goat anti-rabbit IgG (diluted 1:500) at 37° C. The cells were washed again, stained with anti-fluorescence quenching solution containing DAPI, then visualized under a laser scanning confocal microscope (FV-10, Olympus, Japan).

HCN4 (sc-58622) and P16 (A0262) polyclonal antibodies were purchased from Santa Cruz (USA) and Abclonal (China), respectively. CAV3.1 polyclonal antibody (17821-1-AP), TRITC-conjugated goat anti-rat IgG (SA00007-7), FITC-conjugated goat anti-rat IgG (SA00003-11) and CoraLite488-conjugated goat anti-rabbit IgG (SA00013-2) were purchased from Proteintech (China).

### ROS assay

Dihydroethidium (DHE) and 2,7-dichlorodihydrofluorescein diacetate (DCFH-DA) staining were used to assay ROS concentration. For *in vivo* experiments, after quickly frozen, the SAN anatomical position of collected heart tissue was found for serial slice (crosscutting, 4 μm) to prepare frozen sections, followed by the administration of DHE solution (10 μM) and incubation at 37° C for 1 hour. Following a PBS wash-step, sections were examined under a fluorescence microscope. Regarding *in vitro* experiments, mouse atrial myocytes were first seeded into a glass bottom cell culture dish then incubated with a DCFH-DA solution (10 μM) for 1 hour at 37° C. The cells were washed with PBS, then examined under a laser-scanning confocal microscope.

DHE (S0063) and DCFH-DA (S0033S) staining solution were purchased from Beyotime Biotechnology (China).

### Mito-tracker staining

Mito-tracker staining was used to assess mitochondrial activity. Briefly, mouse atrial myocytes were first seeded into a glass bottom cell culture dish, then incubated for 15 minutes with Mito-Tracker Red CMXRos solution (20 nM) at 37° C. The contents were washed with PBS, and the cells were examined under a laser-scanning confocal microscope.

Mito-Tracker Red CMXRos staining solution (C1049B) was purchased from Beyotime Biotechnology (China).

### β-galactosidase staining

Senescence levels were evaluated using a β-galactosidase staining. Summarily, mouse atrial myocytes were seeded into 6-well plates, fixed with β-galactosidase fixative solution for 15 minutes, washed with PBS, then incubated overnight with β-galactosidase staining solution at 37° C. The contents were washed again with PBS, then examined under a microscope.

β-galactosidase staining kit (C0602) was purchased from Beyotime Biotechnology (China).

### Cell cycle assay

Mouse atrial myocytes were digested with trypsin, collected via centrifugation, and fixed for 2 hours with cold ethanol. They were washed with PBS, then incubated with RNase A solution at 37° C for 30 minutes. Next, the cells were incubated with Propidium Iodide solution at 4° C for 30 minutes, washed again with PBS, then subjected to flow cytometry.

RNase A and Propidium Iodide solution (WLA010) were purchased from Wanlei Biotechnology (China).

### CCK-8 assay

Mouse atrial myocytes were seeded onto a 96-well plate at a density of 1×10^4^ cells per well, and five replicate wells were set for each group. The CCK-8 reagent was added to each well to assay for cell proliferation ability. The OD was recorded at 450 nm of each well using a multifunction microplate reader (Multiskan FC, Thermo Fisher Scientific, USA). Blank values from wells containing only DMEM/F12 without cells were subtracted from each reading. The cell proliferation ability of the Control group was set at 100%, and the relative cell proliferation ability of the other groups was calculated normalized to that of the Control group.

CCK-8 reagent (K1018) was purchased from Apexbio Technology LLC (USA).

### Quantitative real-time PCR

Total RNA was isolated using TRIzol reagent and reverse transcribed (42° C for 1 hour and 70° C for 5 minutes) to cDNA using BeyoRT™ M-MuLV reverse transcriptase kit, according to the manufacturer’s instructions, and the cDNA was subjected to RT-qPCR using the fluorescent RT-qPCR kit. Thermocycling conditions were as follows: 94° C for 5 min, followed by 40 cycles of 94° C for 10 s, 60° C for 20 s and 72° C for 30s. Relative gene expression was analyzed using the 2^-ΔΔCt^ method.

BeyoRT™ M-MuLV reverse transcriptase (D7153) and fluorescent RT-qPCR kit (D7268S) were purchased from Beyotime Biotechnology (China). Targeting primers are shown in Table 1 and synthesized by Wanlei Biotechnology (China).

**Table 1:** Primers used for RT-qPCR.

**Gene name**	**Forward primer (5’-3’)**	**Reverse primer (5’-3’)**
*Pitx2*	CGGCAGAGGACTCATTTC	TCCCGCTTTCTCCATTT
*Shox2*	CCCTGGAACAACTCAACGA	ATGACTATCCTGCTGAAATGG
*Cav3.1*	GCCATTGTCACTGTCTTTCA	CCGTTTGCCGATTTCCT
*Hcn4*	AATTCTCCCTAAGGATGTTCG	GATGCCCACGGGTATGA
*β-actin*	CTGTGCCCATCTACGAGGGCTAT	TTTGATGTCACGCACGATTTCC

### Western blotting

After quantified, proteins extracted from SAN or mouse atrial myocytes were separated via sodium dodecyl sulfate polyacrylamide gel electrophoresis. Thereafter, the proteins were transferred to a polyvinylidene difluoride membranes, in a Tris-glycine buffer, and the membranes were blocked with 5% skimmed milk in TBST for 1 hour at room temperature. Next, the membranes were washed with PBS, then incubated overnight with primary antibodies against PITX2, SHOX2, NKX2-5, GATA4, and Histone H3 at 4° C (diluted 1:500). The membranes were washed again with PBS, then incubated for 1 hour with HRP-conjugated affinipure goat anti-rabbit IgG at room temperature (diluted 1:8000). They were washed with TBST, and finally visualized using a chemiluminescence kit.

PITX2 (67201-1-Ig), SHOX2 (16366-1-AP) and NKX2-5 (13921-1-AP) polyclonal antibodies were purchased from Proteintech (China). GATA4 (WL01293), Histone H3 (WL0984a) polyclonal antibodies and HRP-conjugated affinipure goat anti rabbit IgG (WLA023) were purchased from Wanlei Biotechnology (China).

### Electrophysiological assay

hiPSC-CMs were seeded into a CardioExcyte 96 sensor plate at a density of 5×10^4^/well, and cultured in the cardiomyocyte culture medium for 15 days. The cells were plated into different groups, then subjected to various treatments, with the CardioExcyte 96 system (CardioExcyte 96, Nanion Technologies, Germany) used to assay beat rate and field potential duration of hiPSC-CMs.

CardioExcyte 96 sensor plate (NSP-96) was obtained from Nanion Technologies (Germany).

### Statistical analysis

All experiments were repeated at least three times. Data were statistically analyzed using SPSS (version 26.0) and expressed as means ± standard error of the mean (SEM). Comparisons between two and more groups were performed using Student’s t-test and one-way analysis of variance (ANOVA), respectively. Data followed by P<0.05 were considered statistically significant.

## RESULTS

### D-galactose induces senescence in mice

**Figure 2:**
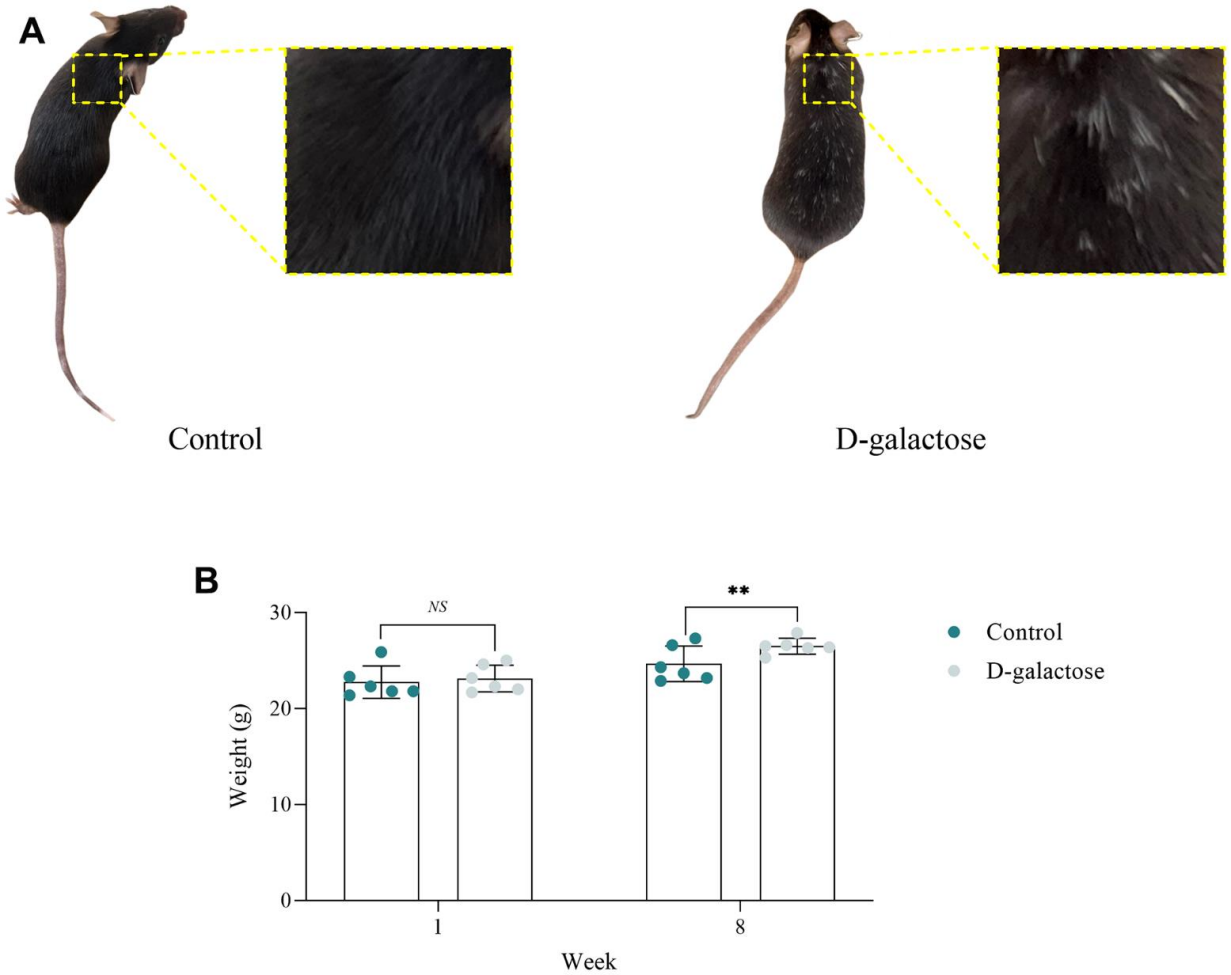
**D-galactose induces senescence in mice.** (**A**) D-galactose causes hair senescence in mice. (**B**) D-galactose causes weight gain in mice. *NS* represents *P*>0.05; ** represents *P*<0.01; Control: control group; D-galactose: D-galactose administered group.

Mice treated with D-galactose exhibited sluggish activity, loss of hair (luster) on their backs and appeared pale. In comparison, those in the Control group were lively and dynamic, with shiny and black back hair (Figure 2A). There were no statistically significant differences between the two groups in terms of weight at week 1, however, mice in the D-galactose group were significantly heavier compared with their counterparts in the Control group at 8 weeks (Figure 2B).

Notably, the decrease in activity and lusterless pale hair suggested that D-galactose induced senescence in mice. Moreover, weight gain suggested that D-galactose induced obesity in mice, which is an important factor that can aggravate oxidative stress and body aging [18].

### D-galactose decreased SAN and heart function in mice

**Figure 3:**
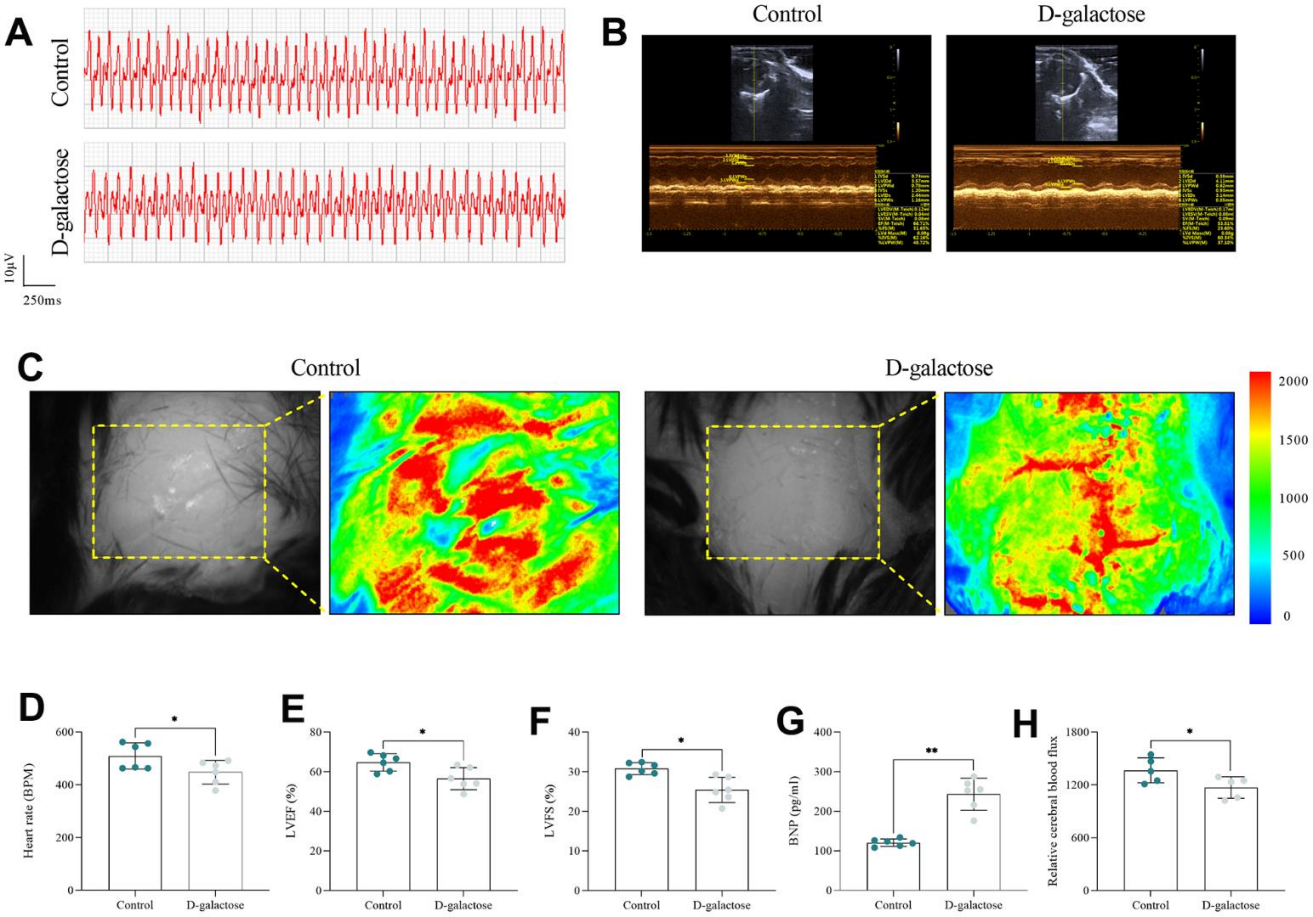
**D-galactose decreased SAN and heart function in mice.** (**A**) Electrocardiogram. (**B**) Echocardiogram. (**C**) Cerebral blood flux. (**D**) Heart rate in the indicated groups. (**E**, **F**) LVEF and LVFS in the indicated groups. (**G**) BNP assay results for the indicated groups. (**H**) Cerebral blood flux results for the indicated groups. * represents *P*<0.05; ** represents *P*<0.01; Control: control group; D-galactose: D-galactose administered group.

After D-galactose administration (at 8 weeks), we used ECG to evaluate SAN function in mice, then analyzed echocardiography, serum BNP concentration and cerebral blood flux to assess cardiac function and organ perfusion in mice. ECG results showed that D-galactose-treated mice had significantly lower heart rates than their counterparts in the Control group (Figure 3A, 3D), indicative of impaired SAN function. Results of echocardiography, and serum BNP (Figure 3B, 3E–3G) as well as cerebral blood flux (Figure 3C, 3H) showed that mice treated with D-galactose had significantly lower left ventricular ejection fraction (LVEF) and left ventricular fractional shortening (LVFS) than those in the Control group, and they exhibited significantly higher and lower content of serum BNP and cerebral blood flux relative to the controls. Collectively, these results suggested that D-galactose treatment caused impairment of cardiac function in mice. Under physiological conditions, sympathetic nervous system should be activated to increase the heart rate and compensate for reduced stroke output. However, results of ECG showed that D-galactose-treated mice had a slower heart rate compared with controls, suggesting that D-galactose can suppress SAN function and cardiac function in mice.

### D-galactose causes fibrosis of sinoatrial node and myocardium

**Figure 4:**
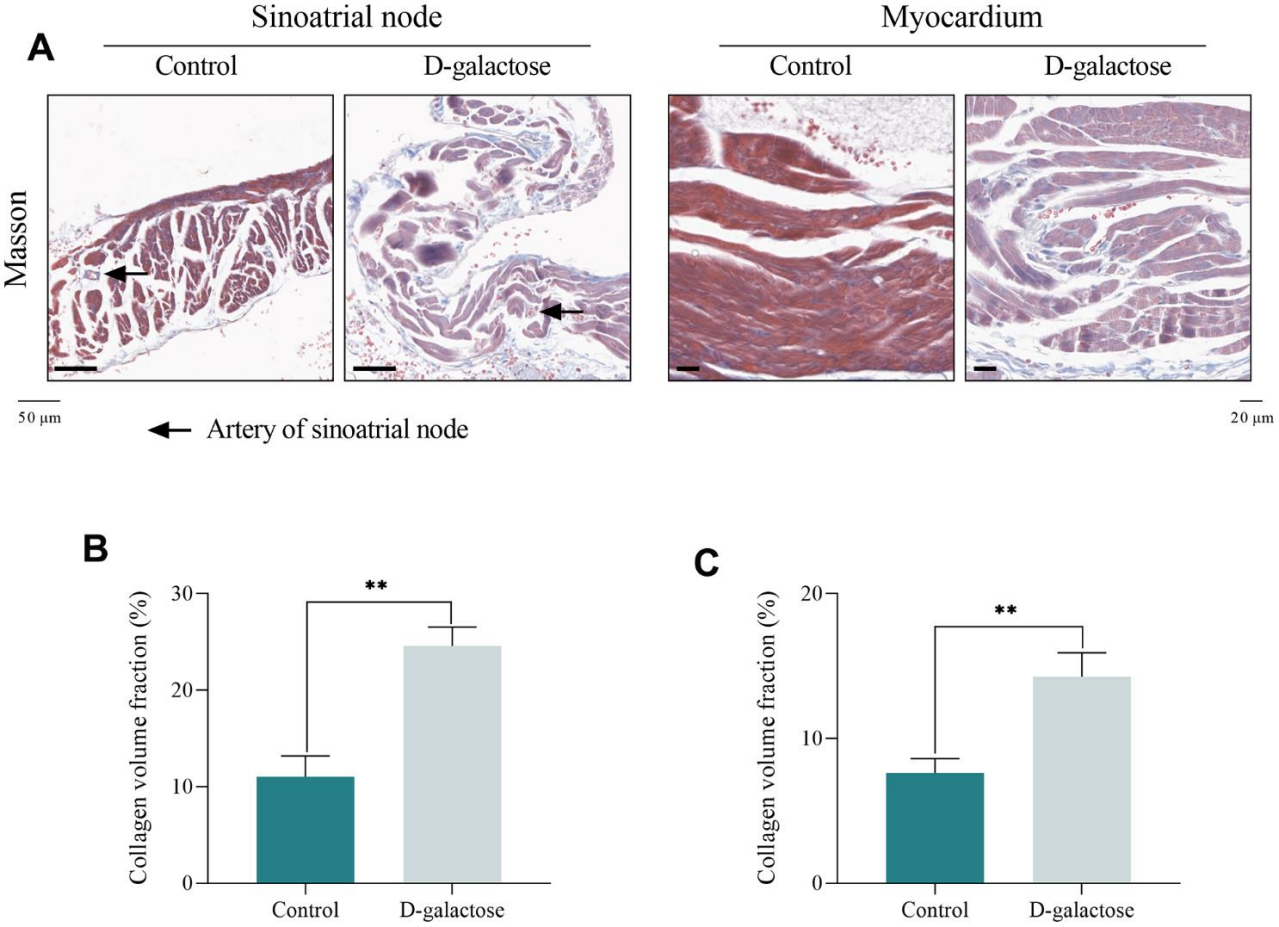
**D-galactose causes fibrosis of sinoatrial node and myocardium.** (**A**) Masson’s trichrome staining of sinoatrial node (Scale = 50 μm) and myocardium (Scale = 20 μm). (**B**) CVF of sinoatrial node. (**C**) CVF of myocardium. Black arrow represents the artery of sinoatrial node; ** represents *P*<0.01; Control: control group; D-galactose: D-galactose administered group.

Fibrotic change is an important characteristic of heart aging, and SAN degeneration often manifests with elevated fibrosis level [19]. In this study, the Masson’s trichrome staining assay was performed to colored fibers, and CVF was analyzed to evaluate the degree of fibrosis. Results of the Masson’s trichrome staining showed that both SAN and myocardium tissue of mice in D-galactose group had a denser blue dyeing than that in Control group (Figure 4A), and CVF analysis demonstrated that mice in D-galactose group had higher fibrosis level compared with those in the Control group (Figure 4B, 4C), and the fibrosis level of SAN was more higher than that of the myocardium. This suggested that D-galactose can cause fibrosis of SAN and myocardium in mice, leading to degenerative changes in the heart.

### D-galactose induces oxidative stress in the sinoatrial node

**Figure 5:**
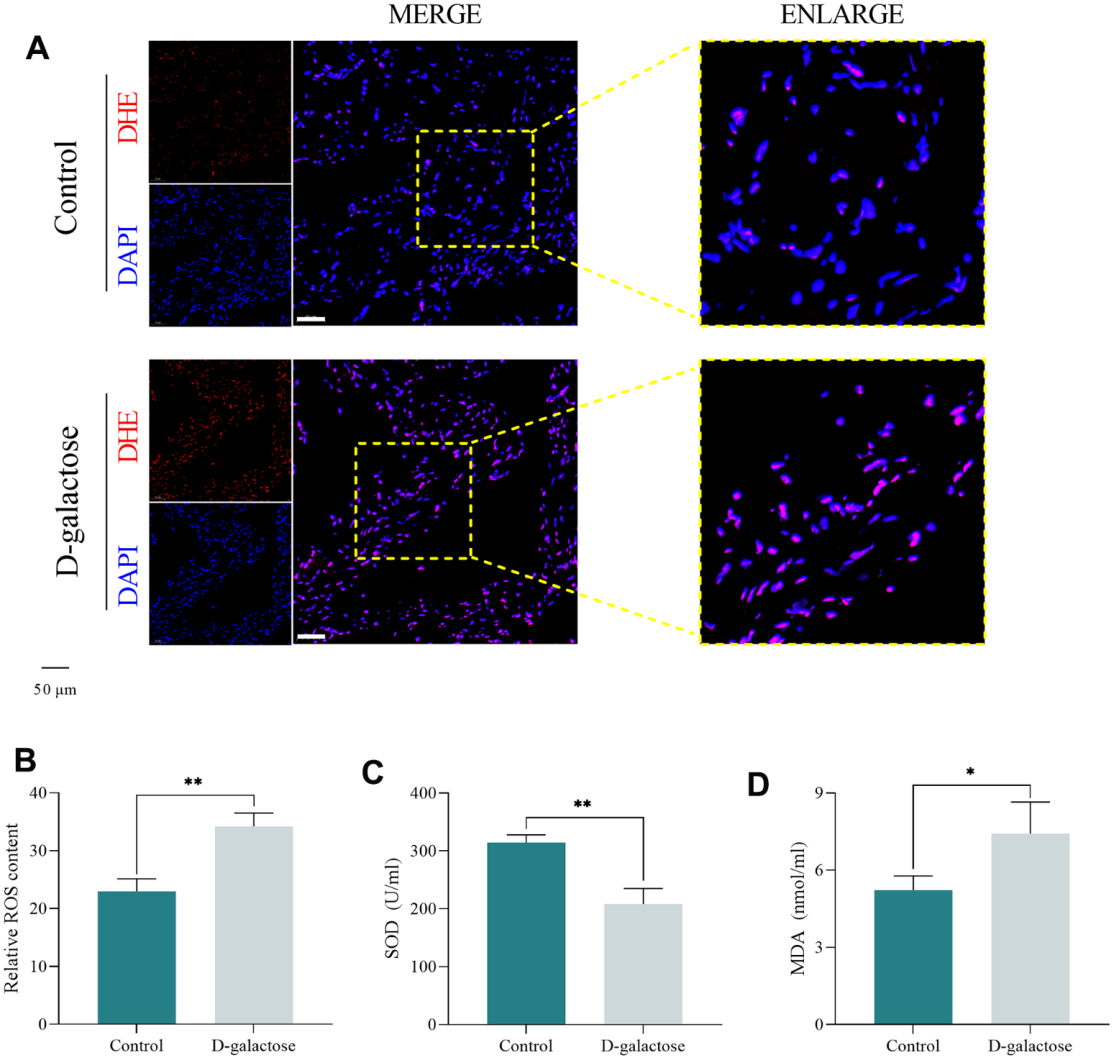
**D-galactose induces oxidative stress in the sinoatrial node.** (**A**, **B**) DHE staining results for the indicated groups. Scale = 50 μm. (**C**, **D**) SOD and MDA assay results for the indicated groups. * represents *P*<0.05; ** represents *P*<0.01; Control: control group; D-galactose: D-galactose administered group.

DHE staining was used to quantify ROS levels in the SAN of mice, while the oxidative status of mice was evaluated by analyzing the concentration of SOD and MDA in serum. DHE staining results showed that mice in the D-galactose group had a significantly higher red fluorescence intensity of the SAN than controls, indicative of ROS accumulation in the SAN (Figure 5A, 5B). Meanwhile, D-galactose-treated mice had significantly lower and higher serum SOD and MDA concentrations, respectively, than those in the Control group (Figure 5C, 5D). These results suggest that D-galactose treatment causes peroxidation in mice, while accumulation of ROS in the SAN may be an important factor causing SND.

### Oxidative stress causes abnormality in the *I_f_* channel

**Figure 6:**
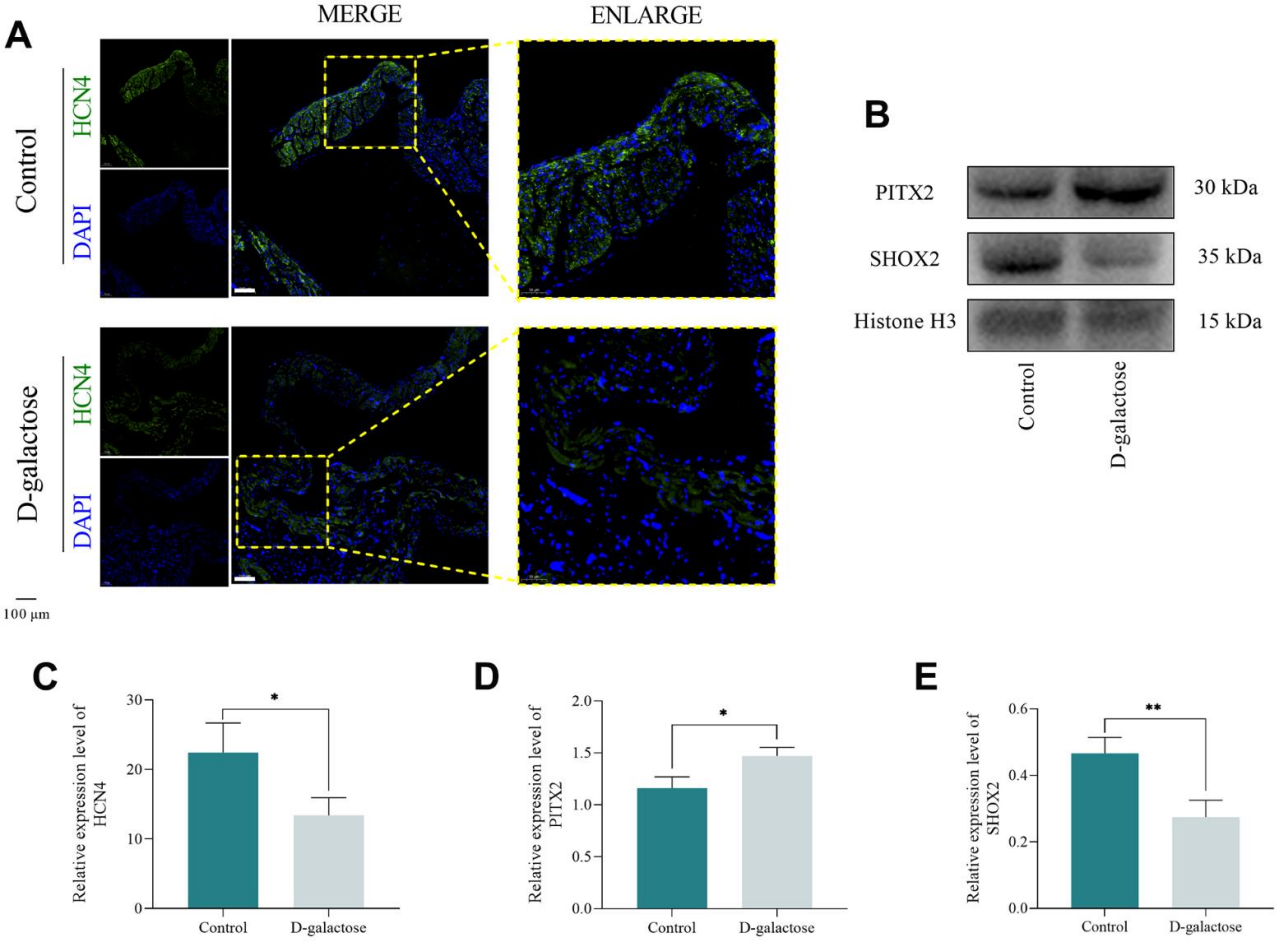
**Oxidative stress causes *I*_f_ channel abnormality.** (**A**) Immunofluorescence staining of the SAN. Scale = 100 μm. (**B**) Western blotting assay. (**C**) Results of immunofluorescence staining. (**D**, **E**) Results of Western blotting assay. * represents *P*<0.05; ** represents *P*<0.01; Control: control group; D-galactose: D-galactose administered group.

Immunofluorescence staining was used to assess the expression of HCN4, while Western blotting was employed to quantify the expression of PITX2 and SHOX2 in mice SAN. Immunofluorescence staining results showed that HCN4 was significantly downregulated in the SAN of D-galactose-treated mice compared to those in the control group (Figure 6A, 6C). Western blots revealed that PITX2 and SHOX2 were significantly upregulated and downregulated, respectively, in the SAN of D-galactose-treated mice relative to those in the control group (Figure 6B, 6D, 6E). These evidences suggest that D-galactose treatment upregulated expression of PITX2 while leading to ROS accumulation in the SAN of mice. Overexpression of PITX2 inhibited normal synthesis of SHOX2, ultimately downregulating HCN4 expression, and impairing the *I*_f_ channel, suppressing pacing function of P cell, causing SND.

### ROS accumulation is responsible for D-galactose-induced senescence

**Figure 7:**
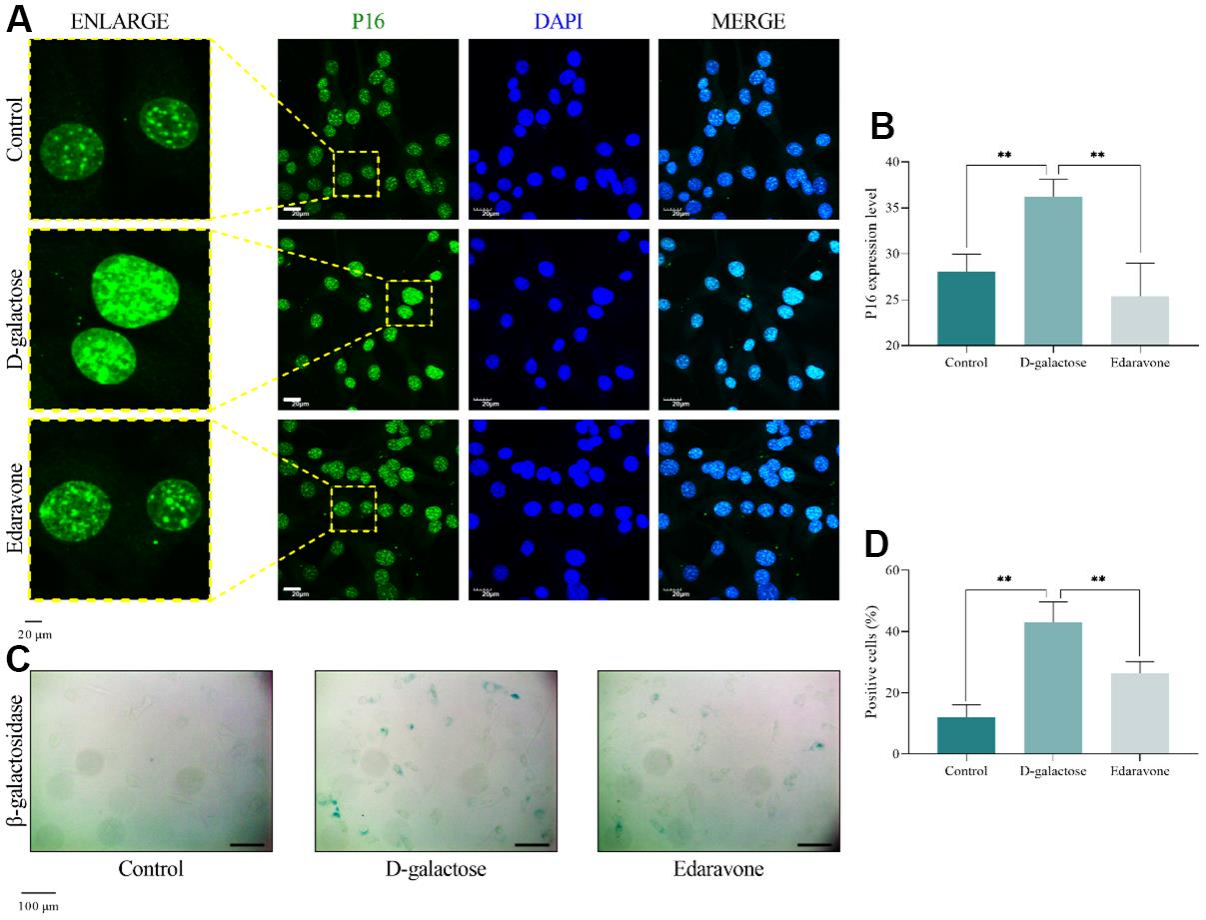
**D-galactose causes cell senescence.** (**A**, **B**) Results of P16 immunofluorescence staining. Scale = 20 μm. (**C**, **D**) Results of β-galactosidase staining. Scale = 100 μm. ** represents *P*<0.01; Control: control group; D-galactose: D-galactose administered group; Edaravone: D-galactose+Edaravone administered group.

Next, we used D-galactose to induce senescence in mouse atrial myocytes, then applied edaravone to scavenge ROS. Thereafter, we assessed the state and function of cells using several approaches including P16 immunofluorescence staining, β-galactosidase staining, Mito-tracker staining, DCFH-DA staining, cell cycle assay, CCK-8 assay, and RT-qPCR. Immunofluorescence and β-galactosidase staining results, showed that cells treated with D-galactose had higher expression levels of P16 and higher enzyme activity of β-galactosidase than controls (Figure 7A–7D), indicative of senescence occurrence. However, edaravone treatment significantly inhibited P16 and β-galactosidase expression (Figure 7A–7D), suggesting that edaravone-mediated ROS scavenging effect improves D-galactose-induced cell senescence, oxidative stress may be an important mechanism for D-galactose-induced cell senescence.

**Figure 8:**
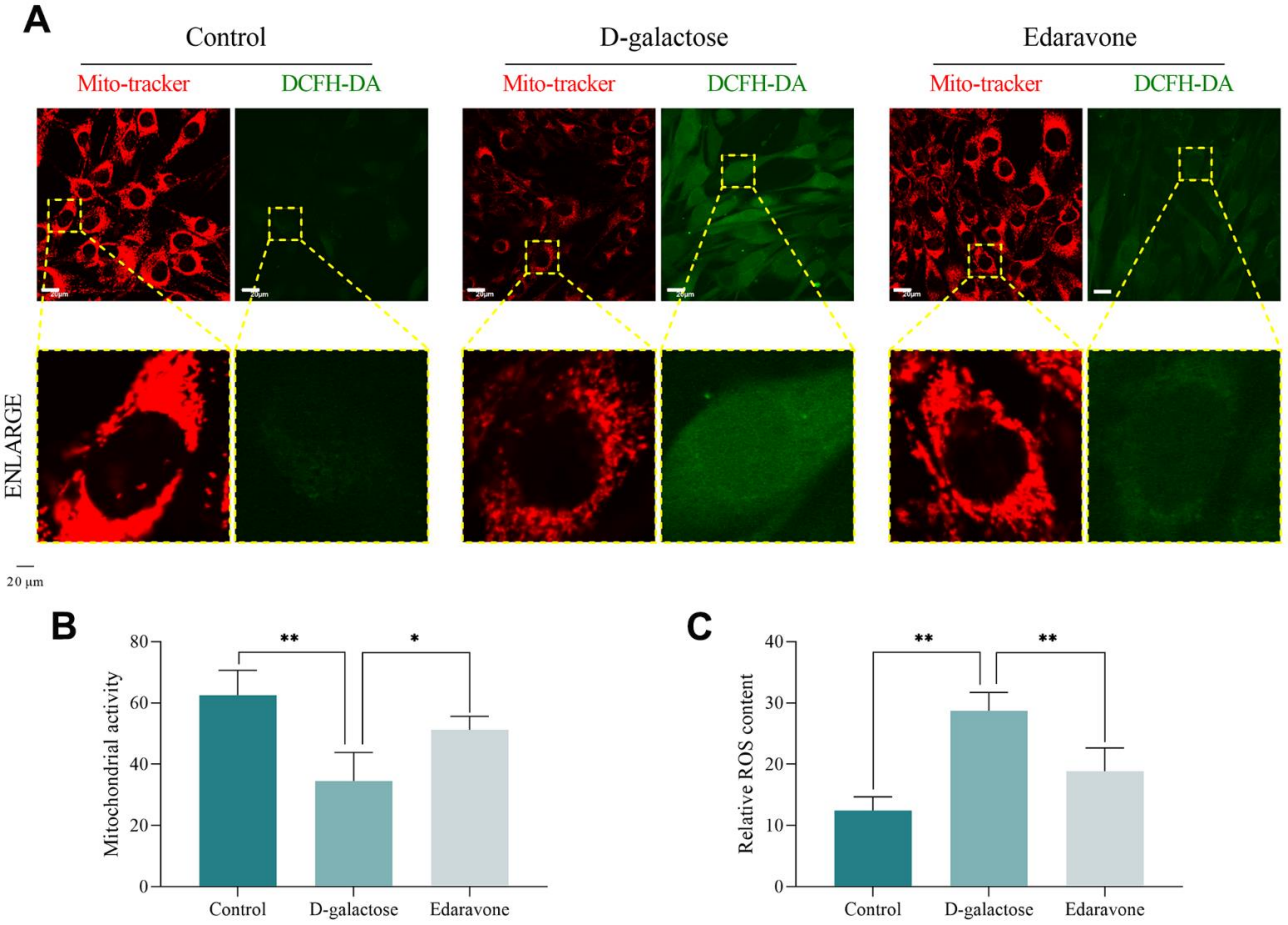
**D-galactose causes cell peroxidation and decreased mitochondrial function.** (**A**) Mito-tracker and DCFH-DA staining results for the indicated groups. Scale = 20 μm. (**B**, **C**) Staining results for the indicated groups. * represents *P*<0.05; ** represents *P*<0.01; Control: control group; D-galactose: D-galactose administered group; Edaravone: D-galactose+Edaravone administered group.

DCFH-DA and Mito-tracker staining results revealed that D-galactose-treated cells had significantly higher ROS concentrations than those in the Control group (Figure 8A, 8C), a phenomenon that was accompanied by mitochondrial dysfunction (Figure 8A, 8B). However, edaravone treatment significantly inhibited D-galactose-induced oxidative stress, suppressed ROS concentration and improved mitochondrial activity (Figure 8A–8C).

**Figure 9:**
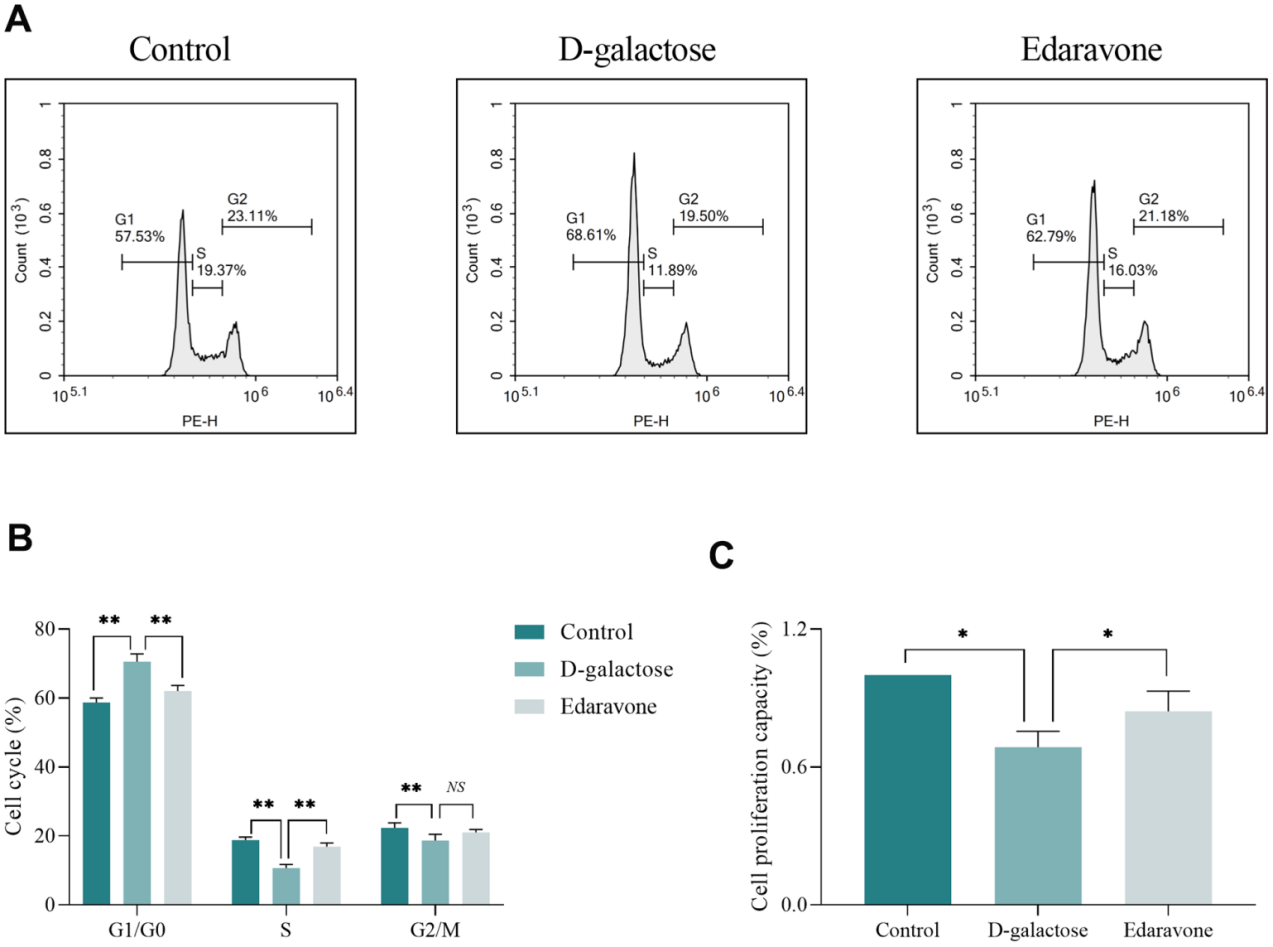
**D-galactose causes cell cycle and proliferation abnormalities.** (**A**, **B**) Results of cell cycle assay. (**C**) CCK-8 assay results for the indicated groups. *NS* represents *P*>0.05; * represents *P*<0.05; ** represents *P*<0.01; Control: control group; D-galactose: D-galactose administered group; Edaravone: D-galactose+Edaravone administered group.

Analysis of the cell cycle assay results revealed that D-galactose treatment increased the proportion of cells in G1/G0 phase, but suppressed that of cells in the S and G2/M phases, compared to the control group (Figure 9A, 9B). On the other hand, CCK-8 assay results revealed that cells in the D-galactose group were characterized by decreased proliferative capacity (Figure 9C). As expected, edaravone treatment not only significantly improved cell cycle abnormalities but also increased cell proliferation (Figure 9A–9C).

**Figure 10:**
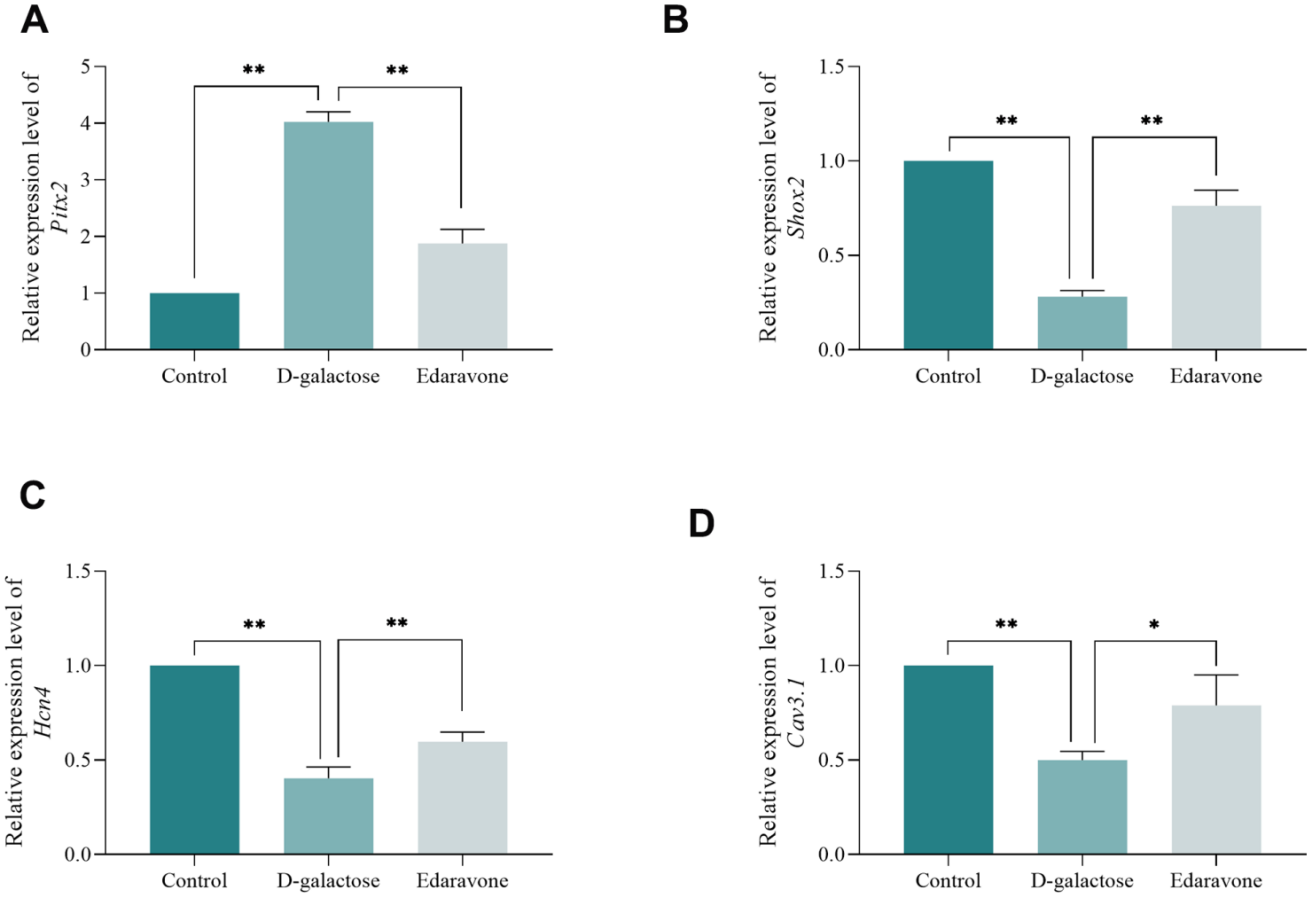
**D-galactose causes abnormal expression of pacing-related gene.** (**A**–**D**) RT-qPCR results for the indicated groups. * represents *P*<0.05; ** represents *P*<0.01; Control: control group; D-galactose: D-galactose administered group; Edaravone: D-galactose+Edaravone administered group.

Collectively, these results confirmed that oxidative stress plays a crucial role in the mechanism underlying D-galactose’s action in induction of senescence. Next, we performed RT-qPCR to quantify expression levels of *Pitx2*, *Shox2*, *Hcn4* and *Cav3.1* mRNA in cells across three groups. Results showed that *Pitx2* was significantly upregulated, while *Shox2*, *Hcn4* and *Cav3.1* were significantly downregulated in D-galactose-treated cells, relative to those in the Control group. However, edaravone treatment not only significantly inhibited expression of *Pitx2*, but also up-regulated that of *Shox2*, *Hcn4* and *Cav3.1* (Figure 10). Collectively, these results suggest that D-galactose induced oxidative stress and caused ROS accumulation in cells, thereby promoting expression of *Pitx2*. Furthermore, overexpression of *Pitx2* inhibited *Shox2* transcription, thereby downregulating expression of downstream *Hcn4* and *Cav3.1*. However, edaravone scavenged ROS, thereby downregulating expression of *Pitx2*, while upregulation that of *Shox2*, *Hcn4* and *Cav3.1*. Taken together, these findings suggested that ROS accumulation may be responsible for D-galactose-induced senescence and pacing dysfunction.

### ROS scavenging and silencing of Pitx2 improves pacemaker ion channel

**Figure 11:**
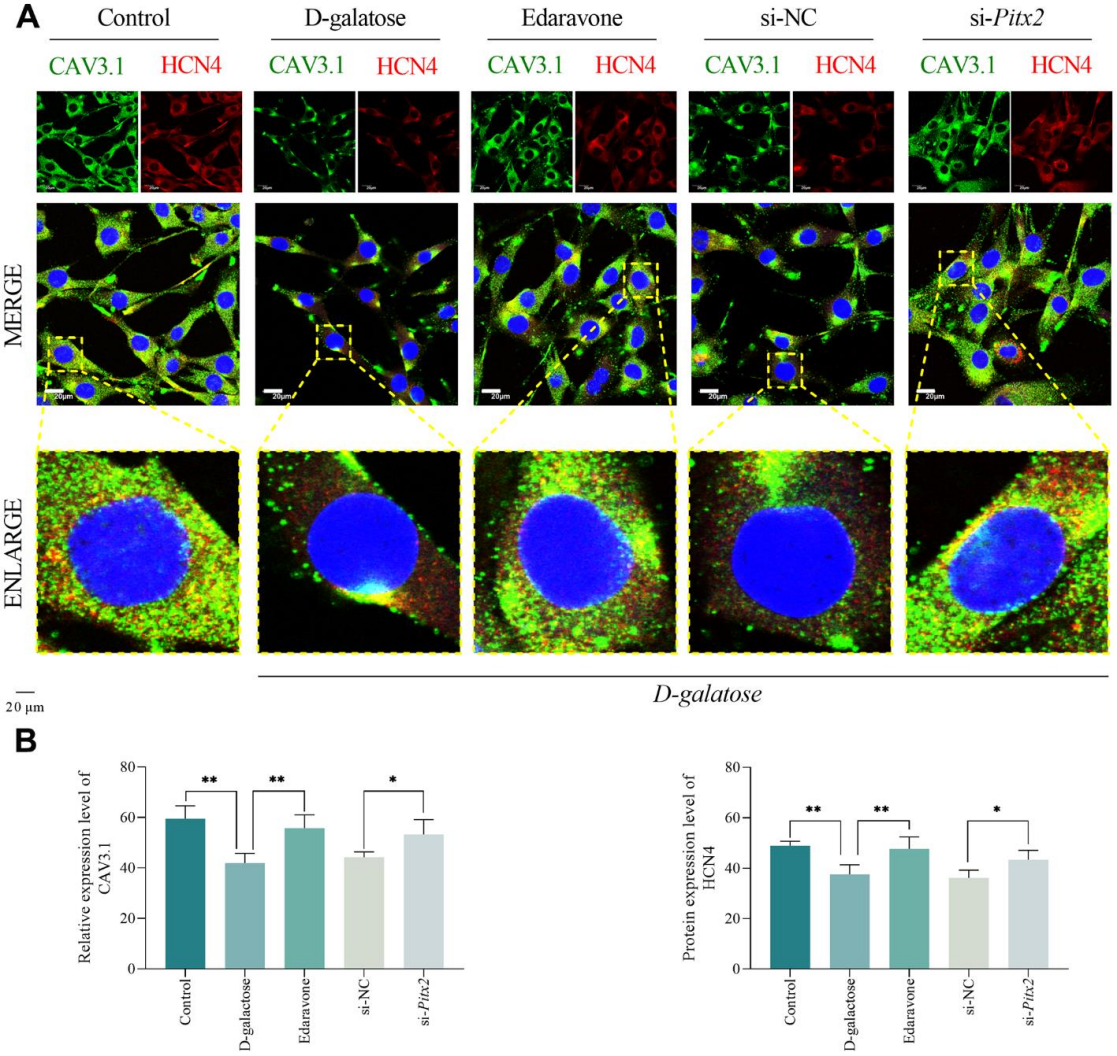
**ROS scavenging and Pitx2 silencing improves pacemaker ion channel.** (**A**, **B**) Immunofluorescence staining results for the indicated groups. Scale = 20 μm. * represents *P*<0.05; ** represents *P*<0.01; Control: control group; D-galactose: D-galactose administered group; Edaravone: D-galactose+Edaravone administered group; si-NC: si-NC transfection+D-galactose administered group; si-*Pitx2*: si-*Pitx2* transfection+D-galactose administered group.

To further verify the role of ROS and *Pitx2*, we used si-*Pitx2* interference sequence to silence *Pitx2* expression, followed by D-galactose treatment, then used immunofluorescence staining to assess expression of pacemaker ion channel factors. Results showed that HCN4 and CAV3.1 were significantly downregulated in cells treated with D-galactose, relative to those in the Control group, whereas edaravone treatment markedly reversed this expression trend. As expected, the expression pattern of HCN4 and CAV3.1 in the si-NC group cells were not different from that in the D-galactose group, however, both genes were significantly upregulated in cells in the si-*Pitx2* group relative to those in the si-NC group (Figure 11A, 11B). Overall, this suggested that D-galactose treatment downregulated HCN4 and CAV3.1 expression, thereby causing dysfunction of *I*_f_ channel and T-type calcium channel, while ROS scavenging and silencing *Pitx2* could interrupt this process.

**Figure 12:**
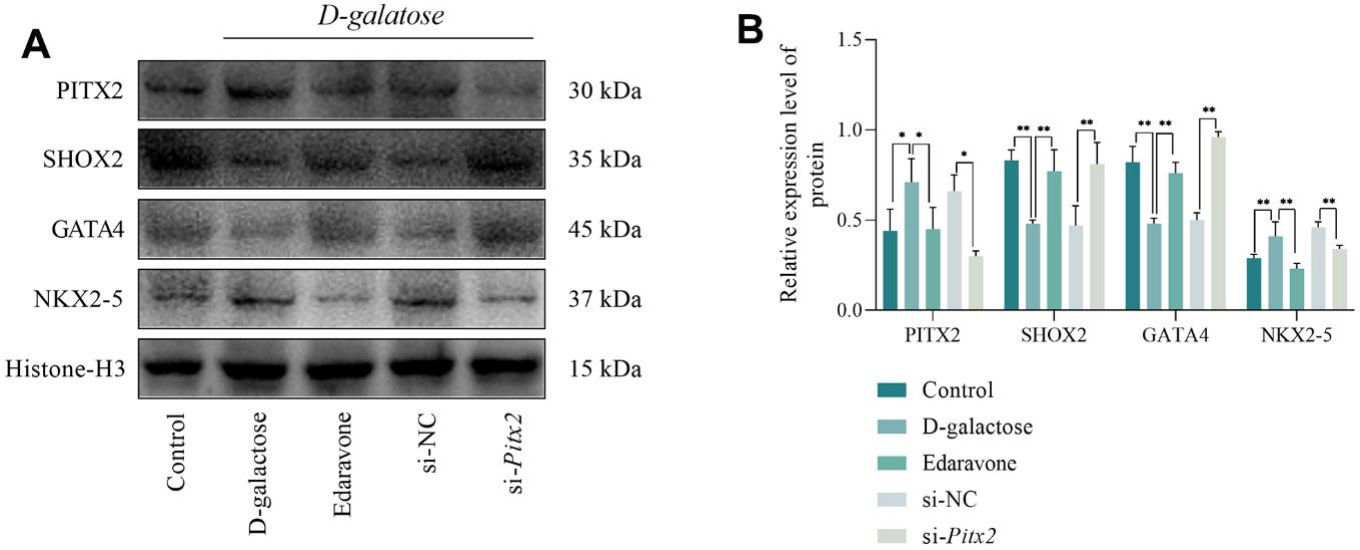
**Results of Western blotting assay.** (**A**, **B**) Results of Western blotting and statistical analysis. * represents P<0.05; ** represents P<0.01; Control: control group; D-galactose: D-galactose-administered group; Edaravone: D-galactose+Edaravone-administered group; si-NC: si-NC transfection + D-galactose-administered group; si-Pitx2: si-Pitx2 transfection + D-galactose-administered group.

To further verify the underlying mechanism of action, we performed Western blotting which revealed that PITX2 and NKX2-5 were significantly upregulated in D-galactose-treated cells compared to levels in the Control group. Notably, this phenomenon was accompanied by downregulation of SHOX2 and GATA4 expression (Figure 12A, 12B). PITX2 and NKX2-5 were significantly downregulated in cells edaravone-treated, relative to D-galactose-treated, while expression of SHOX2 and GATA4 increased significantly (Figure 12A, 12B). There were no significant differences in protein expression between cells in the si-NC and D-galactose groups. However, PITX2 and NKX2-5 were markedly downregulated while SHOX2 and GATA4 were significantly upregulated in the si-*Pitx2* relative to the si-NC group (Figure 12A, 12B). These results confirmed that D-galactose-mediated regulation of SHOX2 expression depends on ROS-mediated PITX2 regulation, while regulation of SHOX2 on HCN4 and CAV3.1 depends on downstream GATA4/NKX2-5 axis.

### ROS scavenging and PITX2 silencing improves pulsatile function in hiPSC-CMs

**Figure 13:**
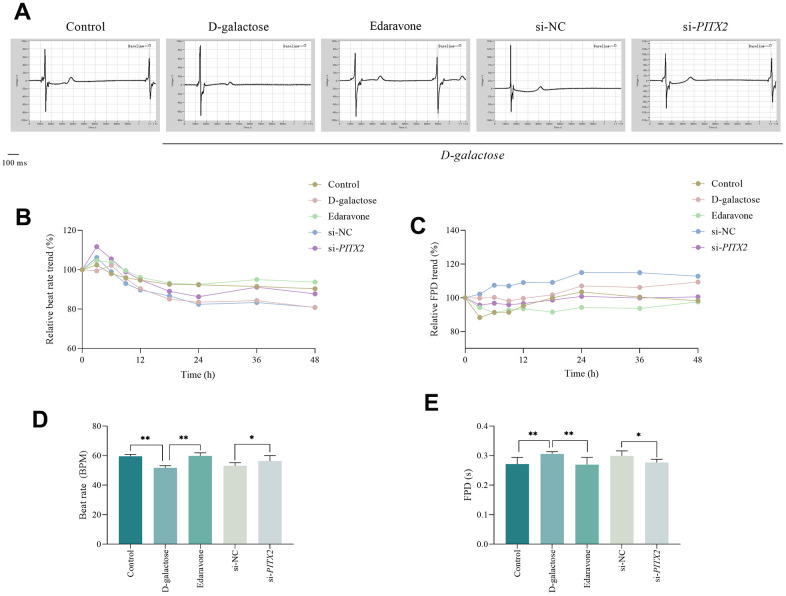
**ROS scavenging and PITX2 silencing improves pulsatile function in hiPSC-CMs.** (**A**) Field potential traces of hiPSC-AMs at 48h. (**B**, **C**) Relative trend of beat rate and field potential duration. (**D**, **E**) Beat rate and field potential duration of hiPSC-AMs at 48h. * represents *P*<0.05; ** represents *P*<0.01; Control: control group; D-galactose: D-galactose-administered group; Edaravone: D-galactose+Edaravone-administered group; si-NC: si-NC transfection+D-galactose administered group; si-*PITX2*: si-*PITX2* transfection+D-galactose-administered group.

To further validate the above findings *in vitro*, we used hiPSC-CMs possessing autonomous rhythm to conduct next electrophysiological experiment. Results showed that prolonging culture time gradually reduced the beat rate in cells across all groups, while gradually prolonging field potential duration. However, cells treated with D-galactose exhibited significantly lower beat rate and longer field potential duration compared to those in the Control group (Figure 13B, 13C). The beat rate of cells in the D-galactose group significantly reduced after 48 hours of treatment, whereas field potential duration was significantly prolonged (Figure 13D, 13E), suggesting that D-galactose treatment caused hiPSC-CMs pulsatile dysfunction. Notably, cells treated with edaravone had higher beat rates and shorter field potential durations compared with those in the D-galactose group (Figure 13D, 13E), suggesting that edaravone treatment improves D-galactose-induced cell pulsation dysfunction. We found no statistically significant differences between the si-NC and D-galactose groups, but cells in the si-*PITX2* group exhibited significantly better beat rates and field potential duration than those in the si-NC group (Figure 13D, 13E).

## DISCUSSION

The incidence of SND increases with senescence in the population, with the elderly being at a higher risk compared to the younger population. Moreover, normal SAN function gradually declines with the increase in age. Thus SAN degeneration is an important factor contributing to the pathogenesis of SND [20, 21]. SAN degeneration often manifests with the characteristics of SAN structural remodeling, such as elevated fibrosis level of the SAN, ROS accumulation of the SAN, and myocardialization of P cells in the SAN, among others. These phenomena may lead to ion channel remodeling and delayed electrical conduction, thereby downregulating the expression of pacing-related ion channels such as HCN4 and T-type calcium channel, which subsequently leads to a decline in SAN pacing function [22, 23].

Funny current (*I*_f_) mediated by *I*_f_ channel and T-type calcium current (*I*_Ca-T_) mediated by T-type calcium channel play a crucial role in maintaining spontaneous depolarization of SAN P cells. Under physiological conditions, *I*_f_ and T-type calcium channels distributed on P cell membrane are activated when the membrane potential reaches the maximum repolarization potential (about -70 mV), while extracellular cations (Na^+^ and Ca^2+^) are transported into P cells. This increases membrane potential to about -40mV, allowing it to reach the threshold potential of L-type calcium channel and producing *I*_Ca-L_ current, which transported Ca^2+^ into P cells, triggered phase-0 depolarization to release one pacing impulse, pacing impulse was then transmitted to peripheral working cardiomyocytes through SAN-transitional cardiomyocytes, to complete the conduction of pacing impulses. However, studies have shown that *I*_f_, *I*_Ca-T_ and *I*_Ca-L_ significantly decrease during senescence of SAN [24]. In our previous study, we demonstrated that HCN4 and CAV3.1 were significantly downregulated in SAN of SND mice induced by natural senescence relative to younger ones, a phenomenon that was accompanied by decreased SHOX2 expression [14, 25]. *Shox2,* a transcription regulator that is highly expressed in SAN, plays a key role in development and differentiation of SAN by regulating downstream *Bmp4*, *Gata4* and *Nkx2-5* [26–28]. Studies have shown that *Shox2*-knockout mice died during pregnancy due to cardiac conduction system defects, including SAN and valve sinus hypoplasia accompanied by the loss of T-box transcription factor 3 and HCN4 expression and ectopic natriuretic peptide A, connexin 40, and NKX2-5 expression in the SAN, which suggested that the differentiation of SAN failed in *Shox2*-knockout mice, and the cells in SAN area differentiated into working cardiomyocytes instead of P cells [29]. The regulation of SHOX2 on downstream BMP4, GATA4 and NKX2-5 is helpful to promote the expression of HCN4 and CAV3.1, which is of great significance for maintaining the pacing function of SAN and inhibiting the transformation of P cells into working cardiomyocytes [30–33].

Here, we used D-galactose to induce senescence in C57BL/6 mice with a view of elucidating its effect on degeneration of SAN, then explored its underlying mechanism of action. *In vivo* results demonstrated that administration of D-galactose induced senescence in young mice, decreased cardiac systolic function and organ perfusion, and elevated BNP levels. Under physiological conditions, the sympathetic nervous system should be activated to increase the heart rate in compensation to counter the decrease of stroke output. However, this has not happened. Although D-galactose suppressed cardiac function in mice, it also caused a decrease in the heart rate, a phenomenon that is similar to chronotropic dysfunction suggesting that SND may have occurred. Next, Masson’s trichrome staining was used to evaluate the fibrosis degree of mice heart and found D-galactose treatment caused significant fibrosis of SAN and myocardium, demonstrating the D-galactose caused degenerative changes in the heart leading to SAN and cardiac dysfunction. Further analyses revealed that D-galactose treatment mediated significant downregulation of HCN4 expression in the SAN of mice. This phenomenon was associated with impairment of the *I*_f_ channel, and decreased *I*_f_, which may be considered the major cause of SND. Analysis of oxidative stress indicators and DHE staining results showed that D-galactose not only induced oxidative stress in mice, but also led to significant accumulation of ROS in the SAN.

To clarify the relationship between oxidative stress and downregulation of HCN4 expression, we quantified levels of PITX2 and SHOX2 in the SAN and found that D-galactose treatment mediated upregulation and downregulation of PITX2 and SHOX2 expression, respectively. *Pitx2,* an upstream suppressor gene for *Shox2*, directly binds to the regulatory sequence of *Shox2* to inhibit SHOX2 expression. Under physiological conditions, *Pitx2* is mainly expressed in the left side of heart, where it plays an essential role in maintaining the “one-sided” expression of *Shox2* [34]. Apart from being a *Shox2* inhibitor, *Pitx2* is also an important regulator of antioxidant stress. Breakdown of the body’s oxidation balance indices *Pitx2* expression to resist oxidation and scavenge ROS [35]. Therefore, we hypothesized that D-galactose induced oxidative stress in SAN, thereby causing ectopic expression of PITX2 in SAN to counteract ROS accumulation. However, high levels of PITX2 inhibited SHOX2 expression, thereby downregulating HCN4 expression via the GATA4/NKX2-5 axis, to finally cause SND.

Next, we confirmed that D-galactose induced cell oxidative stress through assaying ROS concentration and mitochondrial activity, and D-galactose induced cell senescence through assaying the P16 expression, β-galactosidase activity, cell proliferation ability and cell cycle. Then, we used edaravone to eliminate excessive galactose-induced ROS and found that the cell senescence indicators were significantly restored, suggesting that oxidative stress may be an important mechanism underlying D-galactose-induced senescence. RT-qPCR results showed that edaravone significantly downregulated expression of *Pitx2,* but upregulated *Shox2*, *Hcn4* and *Cav3.1*, suggesting that inhibition of oxidative stress could improve expression of *Hcn4* and *Cav3.1* through the *Pitx2*/*Shox2* axis. Next, we evaluated the effect of *Pitx2* by transfecting cells with si-*Pitx2* gene interference sequence and results demonstrated that distribution of *Pitx2* not only promoted SHOX2 expression, but also upregulated HCN4 and CAV3.1 expression through the GATA4/NKX2-5 axis. These results indicated that D-galactose treatment induced oxidative stress, upregulated and downregulated PITX2 and SHOX2 expression, respectively, thus causing subsequent downregulation of HCN4 and CAV3.1 expression through the GATA4/NKX2-5 axis.

To obtain more compelling evidence, we conducted further experiments with hiPSC-CMs which have autonomous rhythm and found that D-galactose treatment caused pulsatile dysfunction of hiPSC-CMs, while scavenging ROS and distribution of *Pitx2* could restore the pulsatile function of hiPSC-CMs. Taken together, these results indicate that D-galactose can effectively induce senescence in both animals and cells, and it is a feasible approach for inducing SAN degeneration and SND. It is also evident that oxidative stress is an important mechanism underlying D-galactose-induced SAN degeneration, while excessive ROS accumulation causes ectopic expression of PITX2, thus downregulating the expression of SHOX2, and cause dysfunction of pacing-related ion channels through the downstream GATA4/NKX2-5 axis, result in SND.
